# Functionally redundant but dissimilar microbial communities within biogas reactors treating maize silage in co-fermentation with sugar beet silage

**DOI:** 10.1111/1751-7915.12308

**Published:** 2015-07-22

**Authors:** Susanne G Langer, Sharif Ahmed, Daniel Einfalt, Frank R Bengelsdorf, Marian Kazda

**Affiliations:** 1 Institute of Microbiology and Biotechnology, Ulm UniversityAlbert-Einstein-Allee 11, 89081, Ulm, Germany; 2 Institute of Systematic Botany and Ecology, Ulm UniversityAlbert-Einstein-Allee 11, 89081, Ulm, Germany

## Abstract

Numerous observations indicate a high flexibility of microbial communities in different biogas reactors during anaerobic digestion. Here, we describe the functional redundancy and structural changes of involved microbial communities in four lab-scale continuously stirred tank reactors (CSTRs, 39°C, 12 L volume) supplied with different mixtures of maize silage (MS) and sugar beet silage (SBS) over 80 days. Continuously stirred tank reactors were fed with mixtures of MS and SBS in volatile solid ratios of 1:0 (Continuous Fermenter (CF) 1), 6:1 (CF2), 3:1 (CF3), 1:3 (CF4) with equal organic loading rates (OLR 1.25 kgVS m^−3^ d^−1^) and showed similar biogas production rates in all reactors. The compositions of bacterial and archaeal communities were analysed by 454 amplicon sequencing approach based on 16S rRNA genes. Both bacterial and archaeal communities shifted with increasing amounts of SBS. Especially pronounced were changes in the archaeal composition towards *Methanosarcina* with increasing proportion of SBS, while *Methanosaeta* declined simultaneously. Compositional shifts within the microbial communities did not influence the respective biogas production rates indicating that these communities adapted to environmental conditions induced by different feedstock mixtures. The diverse microbial communities optimized their metabolism in a way that ensured efficient biogas production.

## Introduction

In 2013, 7.6% of the end energy consumption in Germany was gained from biogas. 48% of the 7850 biogas plants in Germany use renewable raw materials as feedstocks, such as maize silage (MS, 73%) and grass silage (12%) (FNR, 2014).

Utilization in biogas reactors of these plant materials requires retention times of about 100 days even though fibre-rich feedstocks may not get fully degraded (Boe and Angelidaki, 2009; Procházka *et al*., 2012). In contrast, feedstocks containing easy-degradable sugars and alcohols, such as sugar beet silage (SBS) can be rapidly degraded leading to an acidification of the biogas sludge and consequently to an inhibition of methane production (Kryvoruchko *et al*., 2009). Furthermore, SBS is a difficult feedstock in terms of the availability of nutrients and buffering capacity (Demirel and Scherer, 2008). However, it is an interesting co-feedstock for biogas production (Weissbach and Strubelt, 2008) with a similar biogas yield compared with maize silage (Klang *et al*., 2008). Co-fermentation of SBS with other feedstocks can lead to a stable process and has been shown to enhance biogas production resulting in a more efficient biogas process (El-Mashad and Zhang, 2010). However, currently just 2% of the renewable raw materials used in biogas plants in Germany account for SBS (FNR, 2014).

Within the biogas process, methane is mainly produced from hydrogen and carbon dioxide and/or acetate by hydrogenotrophic or acetoclastic methanogenic Archaea (Liu and Whitman, 2008). In general, hydrogenotrophic methanogens are dominant in most agricultural biogas plants especially at high ammonia concentrations (Weiland, 2010). Two members of genera within the methanogenic Archaea, *Methanosarcina* and *Methanosaeta*, can use acetate as substrate for methane formation (Liu and Whitman, 2008). Since these two genera can degrade the same substrate using analogous enzymes, they are defined as functionally redundant in this study. Abundances of different acetoclastic methanogenic species are generally depended on their tolerance towards acetate (Ros *et al*., 2013).

However, an optimal biogas production process relies on a complex and efficient microbial community in order to deal with changing environmental conditions as well as altering substrate compositions. Although, similar archaeal and bacterial groups are present in different biogas reactors, compositions of each of the microbial communities are unique (Sundberg *et al*., 2013). Their identical end-products (CO_2_ and CH_4_) indicate that these microbial communities carry out similar functional processes, regardless of differences in their compositions (functional similarity) (Allison and Martiny, 2008).

In this study, the effect of different feedstock mixtures composed of MS and SBS (CF1, 1:0; CF2, 6:1; CF3, 3:1; CF4, 1:3) on the biogas process was assessed in four continuously fed 12 L lab-scale CSTRs. Therefore, we hypothesized that the biogas production from MS could be improved by co-fermentation of SBS in terms of biogas yield and process stability. Furthermore, in order to correlate possible changes in terms of an improvement of the biogas production, the archaeal and bacterial communities were investigated in relation to different feedstock mixtures by a 454-pyrosequencing approach based on 16S rRNA gene analysis and analysed by rdp (Ribosomal Database Project, Release 11.1; Cole *et al*., 2013).

## Results

### Process performance

All reactors performed well at high yields during the main experimental phase of 80 days at feedstock supply at 1.25 kgVS m^−3^ d^−1^. The cumulative specific biogas yields of all CSTRs (Table 1; Fig. S1) were higher compared with expected values calculated from KTBL data (KTBL 2013, Faustzahlen Biogas).

**Table 1:** Biogas production characteristics of the studied CSTRs. Data recorded for 80 days at OLR of 1.25 kgVS m^−3^ d^−1^

CSTR	CF1	CF2	CF3	CF4	
Feedstock mixture (MS : SBS)	1:0	6:1	3:1	1:3	
Average methane concentration (%)	59 ± 4.3	59 ± 6.2	61 ± 3.9	60 ± 8.2	(*n* = 80)
Cumulative specific biogas yield (l_N_ kg^−1^VS)	755	726	746	799	
Expected specific biogas yield (l_N_ kg^−1^VS)	650	657	662	687	
Avg. biogas production rate (l_N_ h^−1^)	0.39 ± 0.04	0.38 ± 0.06	0.36 ± 0.05	0.41 ± 0.09	(*n* = 80)
pH	7.8 ± 0.15	7.9 ± 0.10	7.9 ± 0.15	7.8 ± 0.10	(*n* = 20)
VFA/TIC ratio	0.06 ± 0.04	0.07 ± 0.04	0.06 ± 0.03	0.07 ± 0.04	(*n* = 20)
C/N ratio	11.1 ± 0.38	11.1 ± 0.24	11.3 ± 0.39	11.4 ± 0.77	(*n* = 5)
NH_4_^+^−N [g l^−1^]	2.7 ± 0.09	2.6 ± 0.010	2.5 ± 0.09	2.1 ± 0.19	(*n* = 4)

l_N_ kg^−1^VS, norm litre per kilogram volatile solids; VFA/TIC ratio, volatile fatty acids/total inorganic carbon ratio; C/N ratio, carbon/nitrogen ratio.

Cumulative specific biogas yields of all reactors differed slightly (Table 1; Fig. S1), whereas the highest average methane concentration (61%) was determined for the 3:1 (MS : SBS) mixture (CF3). Regarding the process dynamics, no substantial differences between the individual mixtures were obvious, while a comparatively higher specific biogas yield (799 l_N_ kg^−1^VS) has been found for CF4 containing the highest rate of SBS, which goes back to slightly higher biogas potential of SBS compared with MS.

Moreover, all reactors showed a stable process performance in terms of pH (7.5–7.9), VFA/TIC (0.04–0.18), C/N ratio (10.6–12.6) and ammonia concentrations (2.1–2.8 g l^−1^) (Table 1).

### Overall phylogenetic analysis

454-amplicon sequencing of the five analysed biogas-producing microbial communities (inoculum and four CSTRs) resulted in a total of 399 258 archaeal and bacterial 16S rRNA gene sequences. The average sequence read length was 713 bp for bacterial and 559 bp for archaeal 16S rRNA gene sequences. The total numbers of sequences per sample obtained by the rdp workflow were given in Table S2 and used for further downstream analysis.

Taxonomic assignment of 16S rRNA gene sequences by the rdp classifier showed a high identification due to the read length of bacterial (760 bp) and archaeal sequences (550 bp). On average, 93% of the bacterial sequences were assigned at phylum level, 61% sequences on family level and 36% sequences at genus level. Furthermore, 99.7% of the archaeal sequences were assigned on phylum level, 89.8% sequences on family level and 87.8% sequences on genus level.

### Species richness and biodiversity

At a sequence homology of 97%, the number of operational taxonomic units (OTUs) based on bacterial 16S rRNA gene sequences was on average 3474 and the Shannon index ranged from 4.0 to 7.4. Data analysis of archaeal 16S rRNA gene sequences resulted in average in 861 OTUs. The respective archaeal Shannon index ranged from 3.1 to 5.1. In general, the estimated numbers of archaeal OTUs as well as the diversity indices were lower compared with the corresponding values for Bacteria (Table S2).

**Figure 1:**
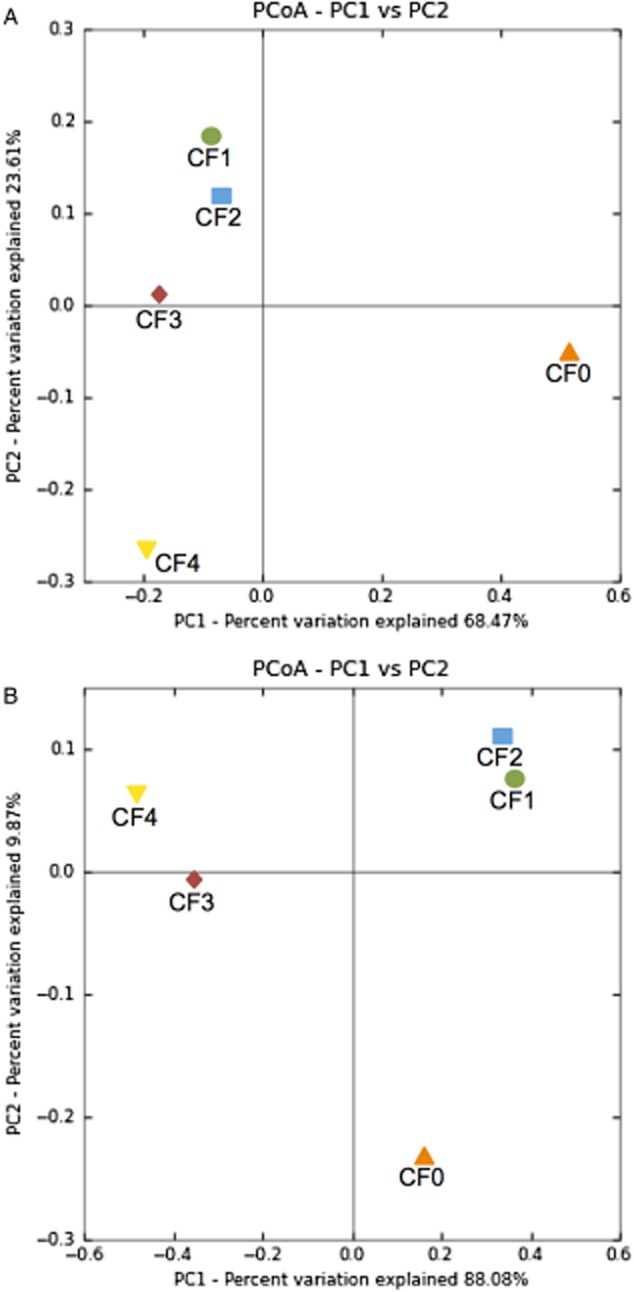
Two-dimensional PCoA of dissimilarities between (A) bacterial and (B) archaeal communities in biogas reactors based on weighted UniFrac analysis. Similar microbial communities are depicted near from each other. PC1 and PC2 explain (A) 92% and (B) 98% of the variation.

Beta-diversity measurements (weighted UniFrac, Table S3) showed that there were high dissimilarities between bacterial communities (Fig. 1A). Compared with the inoculum (CF0), bacterial communities have shifted in similar way in respect to PCoA axis PC1. In comparison with reactor CF1, bacterial communities lined up along PCoA axis PC2 (Fig. 1A).

The archaeal community has also changed compared with the inoculum but at a relatively low extent as the PCoA axis PC2 explains only 9.9% of the total variance (Fig. 1B). The archaeal community in reactor CF1 with MS as mono-substrate was very similar to reactor CF2 with MS and only 14% SBS. Whereas, archaeal communities in reactors CF3 and CF4 with high amounts of SBS were highly dissimilar in comparison to reactors CF1 and CF2.

### Bacterial communities

**Figure 2:**
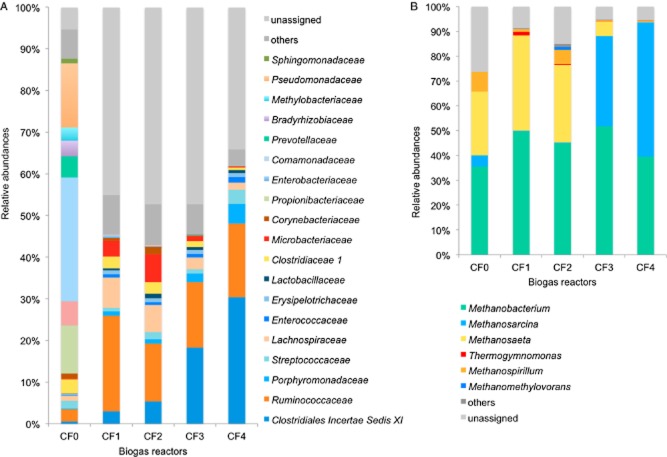
A. Bacterial community composition on family level and (B) archaeal community composition on genus level at the beginning of the experiment in the inoculum (CF0) and after 121 d AD in four lab-scale biogas reactors CF1–CF4 resulting from the 454 amplicon data analysis (confidence cut-off 80%).

The bacterial population in the inoculum was dominated by the phyla *Proteobacteria* (63%) and *Firmicutes* (14%). In all CSTRs, the phylum *Firmicutes* (CF1, 77%; CF2, 70%; CF3, 80%; CF4, 78%) was prevalent. Furthermore, *Bacteroidetes* and *Actinobacteria* were present in the inoculum and all four CSTRs. Relative abundances of members of bacterial families comprising at least 1% of the communities were shown in Fig. 2A. At the start (CF0) of the experiment, the bacterial population was dominated by members of the families *Comamonadaceae* (30%), *Pseudomonadaceae* (15%), *Enterobacteriaceae* (5.8%) (*Proteobacteria*), *Propionibacteriaceae* (12%) (*Actinobacteria*) and *Clostridiaceae* 1 (3.4%) (*Firmicutes*). Only 6.9% of the sequences obtained from sample CF0 remained unassigned. With the addition of feedstock mixtures to the reactors CF1–CF4 over a period of 80 days, bacterial abundances were more unevenly distributed compared with the start (CF0), and the proportion of unassigned sequences was on average 43% (± 6). Furthermore, the bacterial community structure changed completely. Besides *Clostridiaceae* 1, the abundances of the mentioned families found in the inoculum (CF0) were smaller than 1% in all CSTRs (CF1-CF4).

The most dominant families of the domain Bacteria during monofermentation of MS (CF1) were classified as *Ruminococcaceae* (22.8%), *Lachnospiraceae* (7.2%), *Clostridiaceae* 1 (2.8%) within the phyla *Firmicutes* and *Microbacteriaceae* (3.8%) within the phylum *Actinobacteria* (Fig. 2A, marked in yellow-orange tones). With increasing amounts of SBS, the bacterial community shifted gradually, except for *Ruminococcaceae*. Bacteria belonging to the family *Ruminococcaceae* were present in high abundances in all reactors (CF2, 14%; CF3, 16%; CF4, 18%). Most notably, the abundances of members of *Clostridiales Insertae Sedis* XI increased considerably from 3% in reactor CF1 up to 30% in reactor CF4 (CF0, 0.6%; CF2, 5%; CF3, 18%) with the addition of SBS. Furthermore, relative abundances of the families *Streptococcaceae* (CF1, 0.9%; CF2, 1.6%; CF3, 1.1%; CF4, 3.4%), *Enterococcaceae* (CF1, 0.7%; CF2, 0.7%; CF3, 0.8%; CF4, 1.4%) and *Porphyromonadaceae* (CF1, 1%; CF2, 1.1%; CF3, 2.1%; CF4, 4.8%), (Fig. 2A, marked in blue tones) increased slightly with increasing amounts of SBS.

### Archaeal communities

The Archaea detected in all samples belonged to the phyla *Euryarchaeota* (93.3%) and *Crenarchaeota* (6.4%). At the beginning of the experiment (CF0), the archaeal community showed a rather balanced composition between the four genera *Methanobacterium* (35.6%), *Methanosaeta* (25.8%), *Methanospirillum* (7.8%) and *Methanosarcina* (4.5%), while about 25% remained unclassified (Fig. 2B). After anaerobic digestion (AD) for 80 days, the abundances of the hydrogenotrophic *Methanobacteria* (CF1, 50%; CF2, 45%; CF3, 52% and CF4, 40%) in all samples (CF1–CF4) were similar to the start (CF0) independent from the amount of SBS fed to the four CSTRs (Fig. 2B). Furthermore, different members of acetoclastic Archaea were observed in relation to SBS. In reactors fed with MS mainly, *Methanosaeta* species (CF1, 38% and CF2, 32%) dominated. However, with increasing amounts of SBS the archaeal community shifted gradually. In reactors with high amounts of SBS, *Methanosaeta* proportion was as low *as* 6% in CF3 and almost absent in CF4 (0.4%), whereas members of the genus *Methanosarcina* were prevalent in reactors CF3 (36%) and CF4 (54%).

## Discussion

### Process performance

All four lab-scale CSTRs reached high-specific biogas yields (Table 1; Fig. S1). Biogas yields are significantly affected by system technology as well as feedstock quality (KTBL 2013; Mähnert and Linke, 2009). Moreover, high biogas yields are typical for reactors at low organic loading rates (OLRs; Mähnert and Linke, 2009). Biogas production rates and methane concentrations were similar in all CSTRs (Table 1). pH values between 7 and 8, ammonia concentrations below 5 g l^−1^ and low volatile fatty acid/total inorganic carbon (VFA/TIC) ratios (< 0.07 ± 0.04) indicate process stability (Drosg, 2013). Furthermore, C/N ratios were within the optimum range of 10–15 (Weiland, 2010). Consequently, there was no limitation in any of the reactors in terms of nutrients and process instability.

Feedstock characterization (Table S1) showed that SBS contains mainly ethanol, short chain fatty acids as well as sugar, which promote faster feedstock degradation (Weissbach, 2009; Böttcher *et al*., 2011). The comparatively higher specific cumulative biogas yield of reactor CF4 containing the highest rate of SBS (MS : SBS-1:3) goes back to slightly higher biogas potential of SBS (Table 1; Fig. S1). Consequently, the effect of SBS on AD of MS was not quite obvious in terms of biogas yields, methane concentrations and especially in terms of biogas production rates. Thus, the hypothesis of improving biogas production from MS with addition of SBS was rejected.

### Species richness and biodiversity

Results showed a great species richness (OTUs) in all reactors (Table S2). Nelson and colleagues (2011) found similar numbers of archaeal (296 OTUs) and bacterial OTUs (5926) investigating multiple anaerobic digesters by a meta-analysis based on 16S rRNA gene sequencing. However, a high number of OTUs is not only caused by species richness but also by the number of sequence reads obtained by high-throughput sequencing (Roesch *et al*., 2007).

Regarding the total number of species/OTUs, the calculation of the Shannon Index showed that the biodiversity (H′) of the archaeal community was lower compared with bacterial biodiversity in biogas reactors (Table S2). This result is in line with other studies (Fernandez *et al*., 1999; Bengelsdorf *et al*., 2013; Gagliano *et al*., 2015). Differences in feedstock mixtures did not affect the biodiversity notably as indicated by similar values for H′.

### Bacterial communities

CSTRs mainly fed with fibre-rich MS (i.e. CF1 and CF2; Fig. 2A) were dominated by cellulolytic Bacteria such as members of the families *Ruminococcaceae*, *Lachnospiraceae*, *Clostridiaceae* 1 and *Microbacteriaceae*. For example, members of the families *Ruminococcaceae* and *Lachnospiraceae* persist in fibrolytic communities (Brulc *et al*., 2009). They contain a wide range of glycoside hydrolases and carbohydrate-binding modules, enabling them to colonize complex plant material and to degrade recalcitrant polymers, such as cellulose and hemicellulose. Furthermore, they are able to cleave bonds of starch, cellobiose, cellodextrin and chitobiose (Biddle *et al*., 2013). Therefore, members of these families play a common role as active plant degraders (Biddle *et al*., 2013).

In contrast, CSTRs fed with high amounts of SBS were dominated by members of the families *Clostridiales Insertae Sedis* XI, *Porphyromonadaceae*, *Streptococcaceae* and *Enterococcaceae* (Fig. 2A). The family *Clostridiales Insertae Sedis* XI is a heterogeneous group that includes a wide range of different Bacteria (Pagnier *et al*., 2014), and any functional prediction for clostridia only by using phylogenetic information would be highly speculative due to their broad metabolic potential (Fernandez *et al*., 2000). Bacteria of the family *Porphyromonadaceae* are commonly found in mesophilic full-scale biogas plants (Liu *et al*., 2009; Bengelsdorf *et al*., 2013; Li *et al*., 2013; Pope *et al*., 2013). These Bacteria play a central role in glucose fermentation (Li *et al*., 2009). Members of the families *Streptococcaceae* and *Enterococcaceae* are facultative anaerobic, use homolactic acid fermentation (Fisher and Phillips, 2009; Whiley and Hardie, 2009) and were found in other mesophilic biogas reactors (Li *et al*., 2013). Moreover, *Streptococcaceae*-related microorganisms were dominant in biogas reactors fed with high amounts of glucose (Fernandez *et al*., 2000). An increased availability of monosaccharides in CSTRs with high amounts of SBS could have led to the increase of their relative abundances.

Altogether, the composition of each bacterial community was adapted to the supplied feedstock mixture. Thus, functional similarity of highly diverse bacterial communities led to similar biogas production rates in all reactors.

### Archaeal communities

Methane production from acetate is a redundant function performed either by *Methanosaeta* and/or by *Methanosarcina* species (Liu and Whitman, 2008). Due to high affinity to acetate, *Methanosaeta* probably outcompeted *Methanosarcina* in reactors with low amounts of SBS (Fig. 2B). As indicated in Fig. 1B, this pronounced shift took place at the increasing share of SBS addition from 14% (CF2) to 25% (CF3). In reactors with high amounts of SBS, *Methanosarcina* became dominant most likely due to its tolerance to high acetate levels (Ros *et al*., 2013). Consequently, the diverse and functionally redundant members of archaeal communities are able to adapt to changing environmental conditions, and the most competent species consortium is prevailing in the metabolic functions.

### Process performance linked to microbial community structures

Dynamics of microbial communities in biogas reactors depend on several factors for instance on hydraulic retention time, temperature, pH, OLR and ammonia concentration (Dollhopf *et al*., 2001; Ye *et al*., 2007; Rademacher *et al*., 2012; Hai *et al*., 2014; Werner *et al*., 2014). In this study, shifts in microbial communities (Fig. 2) were most likely induced by feedstock compositions varying in the proportions of MS and SBS. With respect to the hypothesis, we showed that these shifts clearly not affect the biogas production rates of CSTRs digesting complex feedstock mixtures (Table 1). A similar result was found by Fernandez and colleagues (2000). They indicated that the functional stability of AD was linked to a flexible microbial community. Moreover, Briones and Raskin (2003) pointed out the importance of diverse and flexible communities for a stable process performance.

In this study, microbial communities carried out functional processes at similar rates, regardless of differences in composition (Fig. S1; Fig. 2A). The individually composed bacterial communities in each CSTR had fulfilled similar functions (functional similarity), which finally resulted in similar biogas production rates in each reactor.

Even more important is the shift in the Archaea composition towards *Methanosarcina* with increasing proportion of SBS. Archaeal communities (Fig. 2B) newly established after SBS co-fermentation are functionally redundant, and thus biogas production rates and methane concentrations (Table 1) were not altered by compositional shifts (Allison and Martiny, 2008).

Functional redundancy is considered as insurance to maintain ecosystem functions under changing environmental conditions (McMahon *et al*., 2007). Moreover, stable ecosystem functions could be sustained by highly dynamic communities (Friedrich *et al*., 2003; Stamper *et al*., 2003; Wittebolle *et al*., 2008; Ayarza *et al*., 2010; Cabrol *et al*., 2012).

### Conclusions

In this study, microbial communities stabilized the digestion process and led to an efficient biogas production from different feedstock compositions. Both bacterial and archaeal communities are responding simultaneously to the supply of easy-to-degrade feedstocks. This finding is unique for the AD research and explains the common observation from biogas practitioners that each biogas plant is an own specific ecosystem, hardly comparable to a second plant.

## Experimental procedures

### Experimental set-up

Anaerobic digestion was performed continuously in four mixed 12 L lab-scale CSTRs (operating volume 10 L, temperature 39°C).

The cylindrical, double-layered biogas reactors made of stainless steel were built in the mechanical workshop of the Ulm University under the supervision of the authors’ institute. Reactor temperature was maintained by passing water from additional hot water bath through copper pipes surrounding the reactors. The water inside the water bath was heated up to 42°C by a heating circulator (JULABO GmbH, Seelbach, Germany).

Feedstocks for biogas production were introduced into the reactors by screw conveyors placed on the bases of each feedstock storage tubes. The corresponding operating unit allowed the subdivision of each rotation into 12 impulses (1 impulse = 30°). Digital card under LabVIEW sent fixed number of impulses to the feeding system at given time interval. In the present experiment, the number of screw conveyor impulses was set individually for each mixture, and the feedstock input was realized automatically on hourly basis. Mixing of SBS with MS might produce high viscosity on the surface of the carrier. Thus, all surfaces were regularly cleaned for good performance as suggested by Scherer and colleagues (2009). The automatic feeding system was controlled daily by the operators of CSTRs. Homogeneous mixing inside CSTRs was achieved by stirring every 10 min for 5 min at 80 r.p.m.

The inoculum originated from a mesophilic (40°C) full-scale biogas plant (Ulm-Gögglingen, Germany) fed with MS (56%), pig manure (30%) and grass silage (14%). Inoculum was used after filtration and kept in the reactors at 40°C for 19 days in order to minimize its background methane production (Li *et al*., 2013; VDLUFA, 2011). Maize silage and SBS were obtained from the above-mentioned biogas plant (Gögglingen, Germany) and the Raiffeisenwarengenossenschaft Emsland-Süd (Lünne, Germany) respectively. Feedstock characteristics (Table S1) make obvious that SBS contains easily degradable components like alcohol, carboxylic acid and sugar, which leads to intensive formation of organic acid that causes low pH values (Böttcher *et al*., 2011). Furthermore, mono-fermentation of SBS requires larger volume of reactors or reduction of OLR and causes foam evolution (Kaiser *et al*., 2008; Böttcher *et al*., 2011).

The underlying aim of the study was to test the influence of different amounts of SBS on the reactor performance. Therefore, the lab-scale CSTRs were fed with different feedstock mixtures of MS and SBS in a ratio (based on volatile solids, VS) of 1:0 in CF1, 6:1 in CF2, 3:1 in CF3 and 1:3 in CF4 respectively. Anaerobic digestion in all CSTRS was started with a low OLR at 0.69 kgVS m^−3^ d^−1^ for a period of 41 days. After the process was stable in terms of biogas yield as well as VFA/TIC values (0.05 ± 0.01), OLR was increased to 1.25 kgVS m^−3^ d^−1^, and the feeding continued for further 80 days.

The experiment was designed for online monitoring of the biogas volume and methane concentrations in each reactor. The volume was assessed by Miligascounter (Ritter GmbH, Bochum, Germany) and given at standard condition (1.013 bar, 0°C and 0% RH). Methane concentrations were measured by infrared sensors (Bluesens GmbH, Herten, Germany, relative accuracy: ± 2% of the measured value). The sensors were calibrated with a 60% CH_4_ certified calibration gas.

Other process parameters like pH, the ratio of VFA/TIC, carbon/nitrogen (C/N) ratio and the amount of total solids (TS) and volatile solids (VS) were determined weekly. Total solids and VS were determined according to APHA methods 2540B and 2540E (APHA, 1999). The pH of the sludge was determined using Metrohm 605 (Filderstadt, Germany). The VFA/TIC ratio was measured by automatic titration (Dosimat 665, Metrohm, Hersau, Switzerland) with 1 M HCl to end-points of pH 5 and 4.3 (Voß *et al*., 2009). The analysis of C and N in the feedstocks and in the sludge was made by TrueSpec C/N analyser (LECO Instrumente GmbH, Mönchengladbach, Germany). Ammonia-nitrogen (NH_4_^+^−N) concentrations were determined with a gas-sensitive ammonia electrode (Type NH 500/2, WTW GmbH, Weilheim, Germany).

Ash-free neutral detergent fibre (NDFom), ash-free acid detergent fibre (ADFom), acid detergent lignin (ADL) and relevant characteristics (crude protein, crude fat, starch, sugar, crude fibre) were determined in the accredited Institute for Oil and Environment, LUFA Nord-West (Oldenburg, Germany). Cellulose, hemicellulose and lignin amounts were obtained from NDFom, ADFom and ADL.

Biogas yields, average methane concentrations, biogas and methane production rates were calculated on hourly basis respectively. Cumulative specific biogas yields were calculated based on cumulative biogas yields for total volatile solid inputs to each individual reactor. Methane concentrations as well as biogas production rates were calculated from average values per day (Table 1).

### Sampling and deoxyribonucleic acid (DNA) extraction

For microbial community analysis, one sample was drawn from the inoculum (CF0) at the beginning of the experiment. Further samples were taken from each reactor (CF1, CF2, CF3 and CF4) after 80 days of AD. Samples were stored at −20°C before DNA was extracted by a modified protocol of Klocke and colleagues (2008). To remove polymerase chain reaction (PCR)-inhibiting substances, 4 ml of each sample were washed with 20 ml sodium phosphate buffer (Hugenholtz *et al*., 2002), 1 ml polyvinylpyrrolidon solution (35%) and 2 ml cetyl trimethylammonium bromide solution (10%) and centrifuged at 5000 r.p.m. for 20 min. The supernatants were removed, and the washing step was repeated 3–4x until the supernatants became clear. After washing, the respective pellets were suspended in 5 ml of a saline EDTA buffer (0.1 mol l^−1^ EDTA, 0.15 mol l^−1^ NaCl), and cells were lysed by Rybolyser (Hybaid, Middlesex, UK) for 3x 45 s at a speed of 6 ms^−1^. Total DNA of the samples was extracted based on phenol/chloroform DNA extraction as described in the protocol of Klocke and colleagues (2008). Deoxyribonucleic acid concentration of the extracted DNA was measured with the NanoDrop 2000 Spectrophotometer (Thermo Fisher Scientific, Wilmingtion, USA) at 260 nm.

### PCR amplification and sequencing

Barcode-tagged primers (Table S4) were used to amplify the V4-V6 region of archaeal 16S rRNA genes (555 bp) and the V3-V6 region of bacterial 16S rRNA genes (760 bp) from the extracted DNA of samples CF0, CF1, CF2, CF3 and CF4 by PCR. Approximately 100 ng of genomic DNA isolated from biogas reactors was used as template for each PCR reaction.

Archaeal 16S rRNA genes were amplified by *ReproFast-*DNA Polymerase (Genaxxon Bioscience GmbH, Ulm, Germany) under the following thermal cycling conditions: a prior denaturation step of 95°C for 5 min, a first loop of 15 cycles of 95°C for 45 s, 55°C for 60 s and 72°C for 60 s, and a second loop of 15 cycles of 95°C for 45 s, 57°C for 60 s and 72°C for 60 s, followed by a terminal elongation step of 72°C for 10 min. Amplification of bacterial 16S rRNA genes for the pyrosequencing approach was done with a DNA-free *Taq DNA Polymerase* (AppliChem GmbH, Darmstadt, Germany) under appropriate buffer conditions. Temperature steps of the PCR were: a prior denaturation step of 94°C for 5 min, a first loop of 10 cycles of 94°C for 60 s, 55°C for 30 s and 72°C for 60 s and a second loop of 20 cycles of 94°C for 30 s, 60°C for 30 s and 72°C for 60 s, followed by a terminal elongation step of 72°C for 10 min. 16S rRNA gene amplification and amplicon length were checked on a 2% agarose gel stained with ethidium bromide. For each sample, three independent PCR reactions were accomplished for either, archaeal and bacterial 16S rRNA genes. Merged PCR amplicons were purified by the purification kit DNA clean & concentrator-5 (ZYMO Research Europe GmbH, Freiburg, Germany). Deoxyribonucleic acid concentration of the purified PCR amplicons was measured with the NanoDrop 2000 Spectrophotometer at 260 nm. Finally, bar code-tagged DNA fragments of bacterial and archaeal 16S rRNA genes were equimolarly merged, and sequencing was performed by Roche GS FLX++ chemistry (MWG eurofins, Berlin, Germany) on 0.5 plate.

### Data analysis

The analysis of 454 amplicon sequencing data was performed by Ribosomal Database Project (RDP). Following the RDP supervised workflow, archaeal and bacterial 16S rRNA gene sequences were sorted according to their bar codes and trimmed. Sequences with a deviation greater than ± 10% from the expected fragment lengths were discarded. Low-quality sequences (minimal quality score 25) were filtered out, and unspecific sequences were excluded by RDP’s sequence selection tool. Remaining sequences were checked for chimeras in the *de novo* mode by Usearch 6.0 (Edgar, 2010). After filtering processes, 55% of the sequences were discarded. Thus, 180 752 sequences remained for downstream analysis. These sequences were aligned by the infernal aligner (Nawrocki and Eddy, 2013). Based on the aligned sequences, complete linkage clustering (farthest neighbour method) was performed with a distance cut-off of 0.03.

The resulting cluster file was used to calculate rarefaction and alpha diversity (Shannon and Chao1 index). Furthermore, differences between microbial communities (ß-diversity) were measured by a weighted UniFrac analysis using qiime (Lozupone *et al*., 2007; Caporaso *et al*., 2010). Therefore, the depth of coverage was adapted to the lowest number of sequences within bacterial or archaeal samples for even sampling depth. A three-dimensional Principal Coordinates Analysis (PCoA) plot based on weighted UniFrac analysis was generated by Emperor for visualization of dissimilarities between microbial communities (Vazquez-Baeza *et al*., 2013).

Representative sequences were picked from the created clusters. Taxonomy was assigned to sequences by the RDP classifier with a confidence cut-off of 80 (Wang *et al*., 2007). Analysis was performed on RDP server infrastructure while data were uploaded. Bacterial and archaeal 16S rRNA gene sequences were deposed in the EMBL database under the study accession number PRJEB7938.

## Supporting Information

**Fig. S1** Specific cumulative biogas yields of reactors CF1–CF4.

**Table S1** Feedstock characteristics of maize and sugar beet silage supplied to CSTRs.

**Table S2** Species richness and diversity estimated from archaeal (Arc) and bacterial (Bac) 16S rRNA gene sequences at 97% homology.

**Table S3** Beta-diversity measurements for bacterial and archaeal communities. The distance of microbial communities was measured between pairs of samples by weighted UniFrac (0 = communities are identical).

**Table S4** Primers for PCR amplification of archaeal and bacterial 16S rRNA genes.
