# Supplementary figures and images for: Functionally redundant but dissimilar microbial communities within biogas reactors treating maize silage in co-fermentation with sugar beet silage
Source: Microb Biotechnol. 2015 Jul 22;8(5):828–36. doi: 10.1111/1751-7915.12308 (PMC4554470; doi:10.1111/1751-7915.12308)

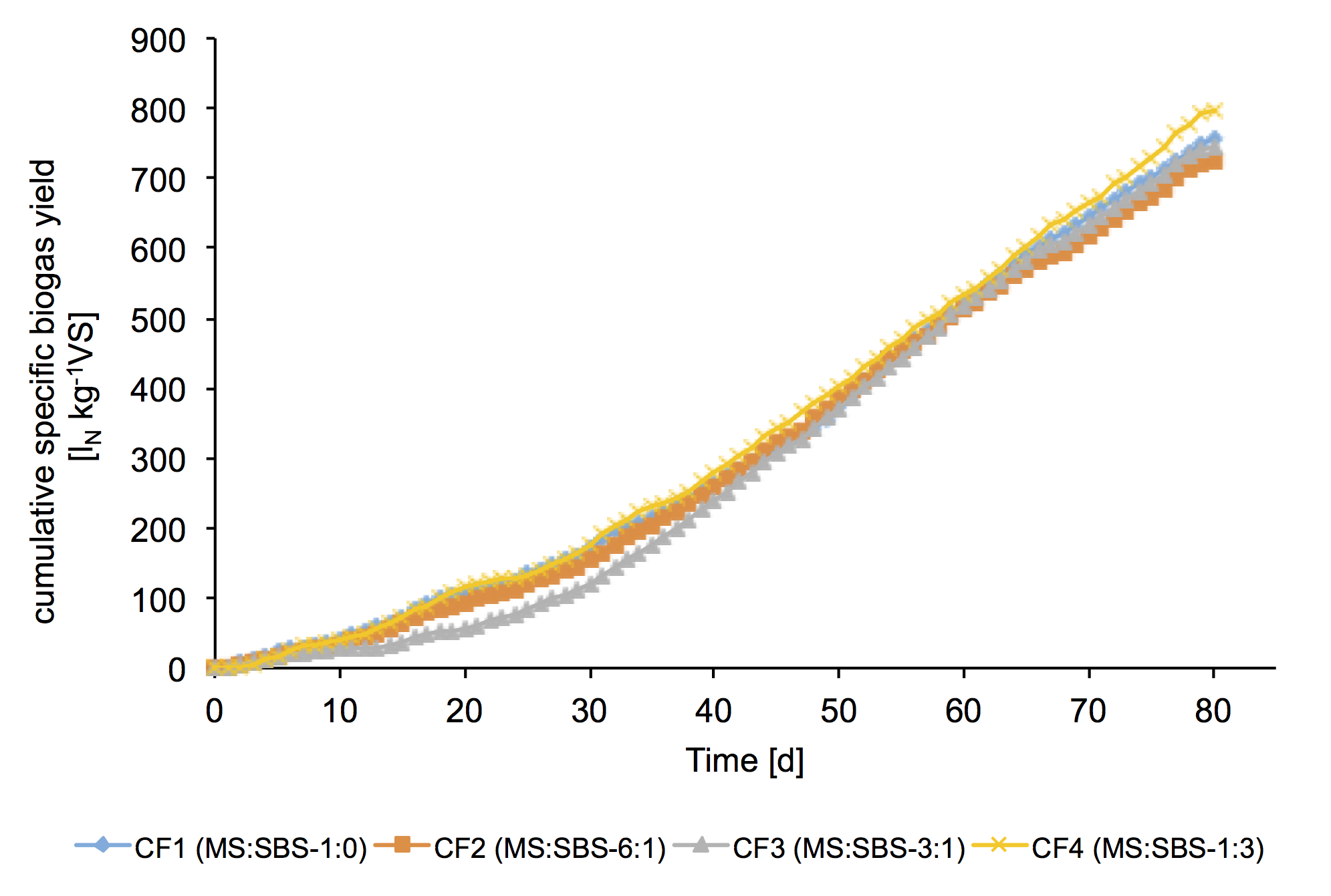

Supplement: Supplementary file 1 [file mbt20008-0828-sd1.tif]
