# Supplementary material for: Functionally redundant but dissimilar microbial communities within biogas reactors treating maize silage in co-fermentation with sugar beet silage
Source: Microb Biotechnol. 2015 Jul 22;8(5):828–36. doi: 10.1111/1751-7915.12308 (PMC4554470; doi:10.1111/1751-7915.12308)
Supplement: Supplementary file 2 [file mbt20008-0828-sd2.docx]

Table S1

| **Parameter** | **Unit** | **Inoculum** | **Maize silage** | **Sugar beet silage** |
| --- | --- | --- | --- | --- |
| pH |  | 8.0 | 5.0 | 3.5 |
| C/N ratio^a^ |  | 12 | 33 | 26 |
| TS^b^ | (% FM^d^) | 6.3 | 36 | 19 |
| VS^c^ | (% TS) | 60 | 80 | 69 |
| Lignin | (g kgTS^-1^) | nd^e^ | 22 | < 9 |
| Cellulose | (g kgTS^-1^) | nd | 160 | 67 |
| Hemicellulose | (g kgTS^-1^) | nd | 158 | 76 |
| Starch | (g kgTS^-1^) | nd | 397 | 74 |
| Sugar | (g kgTS^-1^) | nd | < 5 | 32 |
| Crude fat | (g kgTS-^1^) | nd | 29 | 8.0 |
| Crude protein | (g kgTS^-1^) | nd | 79 | 81 |
| Ethanol | (g kgFM^-1^) | nd | 5.8^f^ | 72 |
| Acetate | (g kgFM^-1^) | nd | 10^f^ | 4.6 |

^a^ C/N ratio, carbon/nitrogen ratio; ^b^ TS, total solids; ^c^ VS, volatile solids; ^d^ FM, fresh mass; ^e^ nd, not determined; ^f^ ethanol and acetate values from the literature according to Weissbach *et al.*, 2008
