# Supplementary material for: Functionally redundant but dissimilar microbial communities within biogas reactors treating maize silage in co-fermentation with sugar beet silage
Source: Microb Biotechnol. 2015 Jul 22;8(5):828–36. doi: 10.1111/1751-7915.12308 (PMC4554470; doi:10.1111/1751-7915.12308)
Supplement: Supplementary file 3 [file mbt20008-0828-sd3.docx]

Table S2

|  | **Sequences** | **Rarefaction**  **[No. of OTUs]** | **Chao1**^a^  **[No. of OTUs]** | **Shannon**^b^ **[H']** | **Coverage [%]** |
| --- | --- | --- | --- | --- | --- |
| **Arc** |  |  |  |  |  |
| CF0 | 937 | 132 | 302 | 3.1 | 44 |
| CF1 | 17,716 | 652 | 889 | 3.8 | 73 |
| CF2 | 36,802 | 1,895 | 2,691 | 5.1 | 70 |
| CF3 | 18,435 | 578 | 803 | 3.9 | 72 |
| CF4 | 46,982 | 1,046 | 1,473 | 4.3 | 71 |
| Ø  **Bac** | 24,174 | 861 | 1,232 | 4.0 | 66 |
| CF0 | 8,300 | 450 | 641 | 4.0 | 70 |
| CF1 | 12,881 | 4,539 | 10,482 | 7.0 | 43 |
| CF2 | 22,051 | 6,895 | 13,744 | 7.4 | 50 |
| CF3 | 6,218 | 2,605 | 6,229 | 6.7 | 42 |
| CF4  Ø | 10,430  11,976 | 2,883  3,474 | 6,219  7,463 | 6.3  6.3 | 46  50 |

^a^ Nonparametric richness estimator based on the distribution of singletons and doubletons.

^b^ A higher number indicates a higher diversity.
