# Supplementary material for: Functionally redundant but dissimilar microbial communities within biogas reactors treating maize silage in co-fermentation with sugar beet silage
Source: Microb Biotechnol. 2015 Jul 22;8(5):828–36. doi: 10.1111/1751-7915.12308 (PMC4554470; doi:10.1111/1751-7915.12308)
Supplement: Supplementary file 4 [file mbt20008-0828-sd4.docx]

Table S3

| **Bac** | **CF0** | **CF1** | **CF2** | **CF3** | **CF4** |
| --- | --- | --- | --- | --- | --- |
| CF0 | 0.00 | 0.65 | 0.62 | 0.70 | 0.74 |
| CF1 |  | 0.00 | 0.19 | 0.27 | 0.46 |
| CF2 |  |  | 0.00 | 0.25 | 0.42 |
| CF3 |  |  |  | 0.00 | 0.33 |
| CF4 |  |  |  |  | 0.00 |
|  |  |  |  |  |  |
| **Arc** |  |  |  |  |  |
| CF0 | 0.00 | 0.37 | 0.39 | 0.57 | 0.74 |
| CF1 |  | 0.00 | 0.09 | 0.72 | 0.89 |
| CF2 |  |  | 0.00 | 0.71 | 0.87 |
| CF3 |  |  |  | 0.00 | 0.24 |
| CF4 |  |  |  |  | 0.00 |
