# Supplementary material for: Functionally redundant but dissimilar microbial communities within biogas reactors treating maize silage in co-fermentation with sugar beet silage
Source: Microb Biotechnol. 2015 Jul 22;8(5):828–36. doi: 10.1111/1751-7915.12308 (PMC4554470; doi:10.1111/1751-7915.12308)
Supplement: Supplementary file 5 [file mbt20008-0828-sd5.docx]

Table S4

| **Primer** | **Sequence 5'-3'** | **Position^a^** | **Reference** |
| --- | --- | --- | --- |
| A0519-F (PARCH519F) | CAGCMGCCGCGGTAA | 519-533 | Ovreas *et al.*, 1997;  Wang *et al.*, 2009;  Klindworth *et al.*, 2013 |
| A1059-R | GCCATGCACCWCCTCT | 1059-1074 | Martin Fischer, Kiel  (AG Schmitz-Streit), personal communication |
| E321-336-F | ACTGAGACACGGYCCA | 321-336 | Wang *et al.*, 2009 |
| E1063-1081-R | CTCACGRCACGAGCTGACG | 1063-1081 | Wang *et al.*, 2009 |

^a^ The numbering of positions is based on *E. coli* 16S rRNA gene
